# National prevalence and clinical impact of pulmonary hypertension in adults listed for lung retransplantation for chronic lung allograft dysfunction

**DOI:** 10.1016/j.jhlto.2026.100651

**Published:** 2026-08-03

**Authors:** Prangthip Charoenpong, Keely Wolf, Mudassir M. Banday, Yasufumi Goda, Eddie Suarez, Cyrus Vahdatpour, Hao Min Pan, Dipaben Dhananjay Modi, Justin Segraves, Muhammad Khyzar Hayat Syed, Andrew S. Potter, Gabriel Loor, Gloria Li, Don Hayes, Ramiro Fernandez, Nirmal Sharma

**Affiliations:** a Department of Medicine, Baylor College of Medicine, Houston, Texas; b Department of Surgery, Baylor College of Medicine, Houston, Texas; c Cincinnati Children’s Hospital Medical Center, University of Cincinnati College of Medicine, Ohio

**Keywords:** pulmonary hypertension, chronic lung allograft dysfunction, pulmonary vascular remodeling

## Abstract

**Background:**

Pulmonary hypertension (PH) has been described in chronic lung allograft dysfunction (CLAD), but its prevalence and prognostic significance in patients listed for lung retransplantation remain uncertain. We sought to determine the national prevalence of PH among adults listed for lung retransplantation for CLAD and to evaluate its association with waitlist and post-transplant outcomes.

**Methods:**

We analyzed SRTR data from May 2005 through September 2025 for adults listed for lung retransplantation for CLAD with right heart catheterization data at listing. PH was defined as mean pulmonary artery pressure (mPAP) greater than 20 mmHg. The primary outcome was waitlist mortality, analyzed using Fine–Gray competing-risk regression with transplantation treated as a competing event. Secondary outcomes included 30-day and 1-year post-transplant mortality.

**Results:**

Among 1440 eligible patients, 58% met hemodynamic criteria for PH at listing. PH prevalence did not differ between bronchiolitis obliterans syndrome (BOS) and restrictive allograft syndrome (RAS). In adjusted competing-risk models, PH was independently associated with increased waitlist mortality (Subdistribution hazard ratio 1.37, 95% CI 1.08-1.74). When mPAP was modeled continuously, higher pulmonary pressures were associated with progressively greater risk of waitlist mortality without a threshold effect. Among patients with complete hemodynamic data, pre-capillary PH was numerically more common in RAS than BOS (31.5% vs 28.2%), while isolated post-capillary PH (10.5% vs 9.9%) and combined pre/post-capillary PH (5.9% vs 7.0%) were similar. PH was not associated with 30-day or 1-year post-transplant mortality. Patients with PH were more likely to remain intubated at 72 hours after retransplantation, while other early postoperative outcomes were similar.

**Conclusions:**

PH is common in adults listed for lung retransplantation for CLAD and is independently associated with increased waitlist mortality, but not early post-transplant mortality.

Lung transplantation remains the definitive treatment for many patients with advanced end-stage lung disease.^1^ Despite improvements in surgical technique, perioperative care, and immunosuppressive management, long-term survival after lung transplantation remains limited.^1^ Chronic lung allograft dysfunction (CLAD) is the leading cause of late graft failure and death after lung transplantation, affecting approximately half of recipients within 5 years and up to three-quarters by 10 years.^2^, ^3^ CLAD, which underwent a refinement of its grading in 2019 by ISHLT consensus, accounts for a substantial proportion of deaths beyond the first post-transplant year and remains the major barrier to durable long-term allograft survival.^4^ CLAD has classically been viewed as an airway-centered disorder, particularly in its bronchiolitis obliterans syndrome (BOS) phenotype.^5^ However, a growing body of work suggests that vascular remodeling is also an important feature of chronic graft failure.

Pulmonary vascular abnormalities, including endothelial injury, intimal hyperplasia, and vascular fibrosis, have been described in CLAD explants and may contribute meaningfully to progressive allograft dysfunction.^6^, ^7^ Pulmonary hypertension (PH) has been reported in patients with CLAD, but prior studies have largely been limited by single-center design, older hemodynamic definitions, or focus on BOS alone.^8^ The clinical significance of PH in patients undergoing evaluation for lung retransplantation remains poorly defined. This question is important because PH may influence both survival while on the waitlist and perioperative risk at the time of retransplantation. A large national registry analysis may therefore clarify both the burden of PH in this population and its relevance to clinical decision-making.

We therefore sought to determine the national prevalence of PH among adults listed for lung retransplantation for CLAD using right heart catheterization (RHC) data recorded at listing and to evaluate its association with waitlist and post-transplant outcomes. We also examined hemodynamic subtypes of PH and assessed whether the burden of pre-capillary and post-capillary PH differed between BOS and restrictive allograft syndrome (RAS).

## Materials and methods

We conducted a retrospective cohort study using data from the Scientific Registry of Transplant Recipients,^9^ including adult patients listed for lung retransplantation for CLAD between May 2005 and September 2025. Follow-up extended through the time of data extraction on 2 February 2026. This study was determined to be exempt from Institutional Review Board review because it utilized de-identified registry data.

### Study population

Adult candidates listed for lung retransplantation for CLAD were eligible for inclusion if RHC data at the time of listing were available. Patients without adequate hemodynamic data to determine PH status were excluded from the primary analysis. Supplementary Figure 1 illustrates the study flow. CLAD phenotype was defined using registry diagnostic codes for lung retransplantation or graft failure. BOS/obstructive phenotype included patients coded as obliterative bronchiolitis, obstructive, or obliterative bronchiolitis obstructive. RAS/restrictive phenotype included patients coded as restrictive or obliterative bronchiolitis restrictive. Because the obliterative bronchiolitis–restrictive code may represent a mixed BOS-restrictive phenotype, this subgroup was examined separately in sensitivity analyses.

### Definition of pulmonary hypertension

PH was defined hemodynamically using RHC data recorded at listing as mean pulmonary artery pressure (mPAP)>20 mmHg.^10^ Hemodynamic variables extracted included systolic, diastolic, and mean pulmonary artery pressures; pulmonary capillary wedge pressure (PCWP); pulmonary vascular resistance (PVR); and, when available, cardiac output. Pre-capillary PH was defined as mPAP greater than 20 mmHg, PCWP 15 mmHg or less, and PVR at least 2 Wood units. Isolated post-capillary PH was defined as mPAP >20 mmHg, PCWP >15 mmHg, and PVR <2 Wood units. Combined pre- and post-capillary PH was defined as mPAP >20 mmHg, PCWP >15 mmHg, and PVR ≥2 Wood units.^10^ Patients with mPAP >20 mmHg but missing PCWP and/or PVR were classified as PH not further classifiable.

### Covariates

Demographic and clinical characteristics were obtained from the registry at the time of listing. Consistent with SAGER guidelines, sex was retained a priori in the primary Fine–Gray model and other adjusted models; however, the purposeful-selection cause-specific Cox sensitivity model was an exception because covariates in that model were selected using a prespecified *p*<0.10 univariable screening threshold. Other covariates included age, race and ethnicity, body mass index, diabetes mellitus, prior malignancy, renal function, Lung Allocation Score (LAS), Composite Allocation Score (CAS), CLAD phenotype (BOS or RAS), and extracorporeal membrane oxygenation (ECMO) support at listing. LAS at listing refers to the score recorded at waitlist registration, whereas LAS at matching refers to the score recorded at donor-recipient matching/allocation, effectively corresponding to the score at transplant among patients who underwent retransplantation. The CAS-era subgroup included patients listed after March 2023, when the CAS replaced the LAS as the lung allocation system in the United States.

### Outcomes

The primary outcome was waitlist mortality following listing for retransplantation. Waitlist mortality was selected as the primary outcome because the study focused on whether PH at listing identifies CLAD retransplant candidates at increased risk before receiving retransplantation. Because transplantation precludes the occurrence of waitlist death, it was treated as a competing event in the primary time-to-event analysis. Secondary outcomes included overall survival from listing and early post-transplant outcomes among patients who underwent retransplantation, including 30- day and 1-year mortality, dialysis, stroke, 72-hour intubation, and airway dehiscence. Airway dehiscence was identified from the registry-reported post-transplant TRR airway dehiscence field, which reflects center-reported coded events. Follow-up time was calculated from listing until death, transplantation, or administrative censoring, whichever occurred first.

### Statistical analysis

Continuous variables were summarized as mean ± standard deviation or median with interquartile range, as appropriate, and categorical variables as counts and percentages. Comparisons between patients with and without PH were performed using Student’s t-test or the Wilcoxon rank sum test for continuous variables and the chi-square or Fisher’s exact test for categorical variables, as appropriate. Missing data were reported for variables included in the descriptive tables and analyses and are summarized in Supplementary Table 11. Patients missing hemodynamic data required to determine PH status were excluded from the primary analytic cohort. Patients with mPAP >20 mmHg but missing PCWP and/or PVR were retained in the PH group but classified as PH not further classifiable for hemodynamic phenotype analyses. Multivariable analyses were performed using complete-case analysis for variables included in each model; observations with missing values for any covariate included in a given multivariable model were excluded from that model, and no multiple imputation was performed.

The primary analysis used multivariable Fine–Gray competing-risk regression to evaluate the association between PH and waitlist mortality, with death as the event of interest and retransplantation treated as the competing event. Subdistribution hazard ratios with 95% confidence intervals were reported. Kaplan–Meier curves from the time of listing were generated for descriptive purposes and compared using the log-rank test, although these estimates were considered descriptive because they do not account for the competing risk of transplantation. Additional unadjusted cumulative incidence analyses were performed to compare waitlist mortality and retransplantation by PH status, treating retransplantation as a competing event for waitlist mortality and waitlist death as a competing event for retransplantation. Retransplantation proportions and time from relisting to retransplantation among retransplanted patients were compared by PH status.

Multivariable Fine-Gray models were adjusted a priori for clinically relevant covariates, including age, sex, ethnicity, CLAD phenotype, body mass index, diabetes, renal function, and ECMO support at listing. As a sensitivity analysis, cause-specific Cox proportional hazards modeling was performed with transplantation treated as a censoring event. The proportional hazards assumption was assessed using Schoenfeld residuals and global tests of proportionality, and no meaningful violations were identified. To assess whether the association between mPAP and waitlist mortality was nonlinear, a restricted cubic spline with 4 knots was first fit in a cause-specific Cox model, with retransplantation treated as a censoring event. Because there was no evidence of nonlinearity, mPAP was modeled linearly in the primary Fine–Gray subdistribution hazard model and reported per 1-mmHg increase.

Additional sensitivity analyses evaluated PVR as both a continuous and categorical predictor of waitlist mortality using Fine–Gray competing-risk regression. PVR categories were defined as <2 WU, 2 to <3 WU, and ≥3 WU, with PVR <2 WU used as the reference group. We also performed a sensitivity analysis using mutually exclusive hemodynamic phenotypes, with no PH as the reference group. Prespecified subgroup analyses were conducted by CLAD phenotype. Among patients who underwent retransplantation, multivariable logistic regression was used to evaluate factors associated with 30-day and 1-year post-transplant mortality. All statistical tests were two-sided, and a *p*-value <0.05 was considered statistically significant. Analyses were performed using R version 4.5.2.

## Results

### Study cohort and prevalence of pulmonary hypertension

A total of 1714 adults listed for lung retransplantation for CLAD were included in the overall cohort, including 1371 (80.0%) with BOS and 343 (20.0%) with RAS. Hemodynamic data sufficient to determine PH status were available for 1440 patients (84.0%), who comprised the analytic cohort; 274 patients (16.0%) were excluded because PH status could not be determined due to incomplete hemodynamic data (Supplementary Figure 1). Among those, 837 (58.1%) met hemodynamic criteria for PH (mPAP >20 mmHg), whereas 603 (41.9%) did not. The prevalence of PH did not differ significantly by CLAD phenotype: PH was present in 58.3% of patients with BOS and 57.4% with RAS (*p* = 0.766) (Supplementary Table 10), indicating similar PH burden across phenotypes. Patients with and without PH were similar in age and did not differ significantly with respect to sex, race, and ethnicity, diabetes mellitus, prior malignancy, or renal function at the time of listing. However, compared with those without PH, patients with PH had a higher body mass index, higher LAS, and greater use of oxygen supplementation at listing, consistent with greater clinical severity at listing (Table 1). In sensitivity analyses separating pure restrictive CLAD from mixed obstructive-restrictive coding, PH prevalence, and hemodynamic phenotype distribution were similar across BOS/obstructive, pure restrictive, and mixed obstructive-restrictive phenotype groups (Supplementary Table 8).

**Table 1:** Baseline Characteristics of CLAD Patients Listed for Lung Retransplantation According to Pulmonary Hypertension Status

**Characteristic**	**CLAD without PH (n = 603)**	**CLAD with PH (n = 837)**	***p*-value**
*Demographics*			
Age at listing, years	45.2±14.1	46.2±14.6	0.196
Female sex, n (%)	287 (47.6)	394 (47.1)	0.887
Hispanic ethnicity, n (%)	57 (9.5)	88 (10.5)	0.568
*Race/ethnicity, n (%)*			0.592
White	488 (81.2)	653 (78.2)	
African American	38 (6.3)	69 (8.3)	
Hispanic ethnicity, n (%)	57 (9.5)	88 (10.5)	
American Indian/Alaska Native	15 (2.5)	18 (2.2)	
Pacific Islander	1 (0.2)	4 (0.5)	
Multiracial	2 (0.3)	3 (0.4)	
*Clinical characteristics*			
Body mass index, kg/m²	21.8±7.6	24.0±7.5	<0.001
Current or prior smoking, n (%)	166 (27.5)	285 (34.1)	0.010
Diabetes, n (%)	204 (33.9)	308 (37.4)	0.578
Creatinine, mg/dL	1.08±0.67	1.14±0.70	0.123
Serum albumin, g/dL	3.70±0.68	3.75±0.61	0.490
Receiving oxygen supplementation at listing, n (%)	366 (74.4)	537 (79.6)	0.037
Oxygen requirement, L/min	5.6±6.5	5.3±5.8	0.451
ECMO support at listing, n (%)	6^1^	18 (2.2)	0.139
*Disease characteristics*			
CLAD phenotype (BOS), n (%)	473 (78.4)	662 (79.1)	0.816
Lung Allocation Score at listing	44.2±14.3	49.2±16.7	<0.001
Lung Allocation Score at matching	52.9±20.2	56.6±21.5	0.003

Values are presented as mean ± SD or n (%). Lung Allocation Score at listing refers to the score recorded at waitlist registration. Lung Allocation Score at matching refers to the score recorded at donor-recipient matching/allocation, effectively corresponding to the score at transplant among patients who underwent retransplantation. BMI, body mass index; BOS, bronchiolitis obliterans syndrome; CLAD, chronic lung allograft dysfunction; PH, pulmonary hypertension

### Hemodynamic phenotype of pulmonary hypertension

Hemodynamic phenotypes were classified as mutually exclusive categories and are summarized overall and by CLAD phenotype in Supplementary Table 10. Overall, 837 patients (58.1%) met criteria for PH, including 416 (28.9%) with pre-capillary PH, 144 (10.0%) with isolated post-capillary PH, 97 (6.7%) with combined pre- and post-capillary PH, and 180 (12.5%) with PH not further classifiable because of missing PCWP and/or PVR. Pre-capillary PH was numerically more common in RAS/restrictive than BOS/obstructive phenotype (31.5% vs 28.2%), whereas isolated post-capillary PH (10.5% vs 9.9%) and combined pre- and post-capillary PH (5.9% vs 7.0%) were similar between phenotypes. RHC findings are summarized in Table 2. As expected, patients with PH had significantly higher pulmonary artery pressures than those without PH, with a mPAP of 26.9 ± 5.8 mmHg versus 16.5 ± 3.0 mmHg (p < 0.001). PCWP was also higher in the PH group (13.3 ± 5.4 vs 7.8 ± 3.7 mmHg; *p* < 0.001). In addition, patients with PH had higher pulmonary vascular resistance and modestly higher cardiac output than those without PH (2.69 ± 1.56 vs 1.89 ± 0.96 Wood units, and 5.50 ± 1.73 vs 4.97 ± 1.42 L/min, respectively; both *p* < 0.001). In sensitivity analyses separating pure restrictive CLAD from mixed obstructive-restrictive coding, hemodynamic variables were similar across BOS/obstructive, pure restrictive, and mixed obstructive-restrictive phenotype groups (Supplementary Table 8).

**Table 2:** Hemodynamic Characteristics From Right Heart Catheterization at Listing

**Variable**	**CLAD without PH**	**CLAD with PH**	***p*-value**
sPAP	26.6±5.1	39.5±8.5	<0.001
dPAP	10.1±4.2	18.4±5.8	<0.001
mPAP	16.5±3.0	26.9±5.8	<0.001
PCWP	7.8±3.7	13.3±5.4	<0.001
CO	5.0±1.4	5.5±1.7	<0.001
PVR	1.89±0.96	2.69±1.56	<0.001

Values are presented as mean ± SD. CO, cardiac output; dPAP, diastolic pulmonary artery pressure; mPAP, mean pulmonary artery pressure; PCWP, pulmonary capillary wedge pressure; PVR, pulmonary vascular resistance; sPAP, systolic pulmonary artery pressure

### Pulmonary hypertension and waitlist mortality

**Figure 1:**
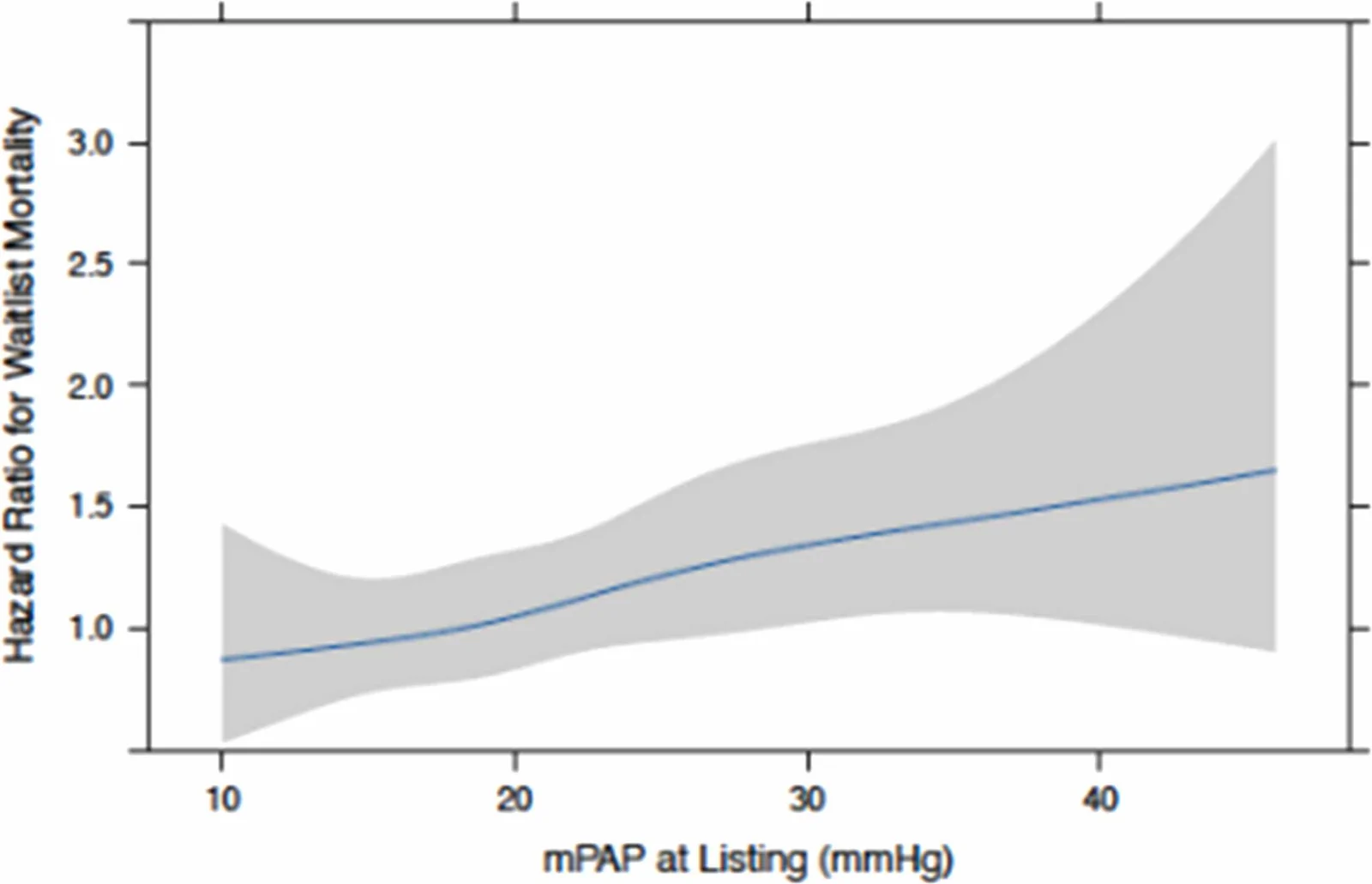
Association Between Mean Pulmonary Artery Pressure and Waitlist Mortality: Restricted cubic spline analysis demonstrating the association between mPAP at listing and the risk of waitlist mortality among CLAD retransplant candidates. The spline curve represents the adjusted hazard ratio from the cause-specific Cox model used to assess the functional form of the association between mPAP and waitlist mortality, with lung transplantation treated as a censoring event. Shaded areas indicate 95% confidence intervals. There was no evidence of a nonlinear relationship between mPAP and waitlist mortality (p for nonlinearity = 0.90), suggesting a progressive increase in risk with higher pulmonary artery pressures without a clear hemodynamic threshold. CLAD, chronic lung allograft dysfunction; mPAP, mean pulmonary artery pressure

In adjusted Fine–Gray competing-risk models, PH was independently associated with increased waitlist mortality (sHR 1.37, 95% CI 1.08-1.74; *p*=0.009) (Table 3**)**. In an additional sensitivity model using the three-group CLAD phenotype classification, PH remained independently associated with waitlist mortality (sHR 1.37, 95% CI 1.08-1.73; *p*=0.010; Supplementary Table 9). This association persisted despite adjustment for clinically relevant covariates, indicating that PH captures risk beyond that reflected by standard listing severity measures. Hispanic ethnicity was also associated with lower waitlist mortality in the adjusted Fine–Gray model (sHR 0.62, 95% CI 0.40-0.97; *p*=0.038). When mPAP was evaluated as a continuous variable, there was no evidence of a nonlinear association with waitlist mortality (p for nonlinearity = 0.90). In linear Fine–Gray analysis, each 1 mmHg increase in mPAP was associated with a modest but significant increase in waitlist mortality risk (sHR 1.02, 95% CI 1.01-1.04, *p* = 0.008), consistent with a progressive increase in risk across the range of pulmonary pressures rather than a discrete threshold effect (Figure 1).

**Table 3:** Multivariable Fine–Gray Competing-Risk Regression for Waitlist Mortality Among CLAD Retransplant Candidates

**Variable**	**Subdistribution Hazard Ratio (sHR)**	**95% CI**	***p*-value**
Pulmonary hypertension	1.37	1.08-1.74	0.009
Age (year)	0.99	0.99-1.00	0.216
Female sex	0.88	0.70-1.11	0.277
Body mass index (kg/m²)	0.98	0.96-1.01	0.208
Hispanic ethnicity	0.62	0.40-0.97	0.038
BOS phenotype (vs RAS)	1.03	0.78-1.37	0.839
Creatinine (mg/dL)	0.94	0.77-1.13	0.497
Diabetes mellitus	0.88	0.70-1.10	0.265
ECMO support at listing	0.33	0.08-1.39	0.130

Fine–Gray competing-risk regression was used to evaluate waitlist mortality with lung transplantation treated as a competing event. Models were adjusted for clinically relevant covariates selected a priori. BOS, bronchiolitis obliterans syndrome; CLAD, chronic lung allograft dysfunction; ECMO, extracorporeal membrane oxygenation; RAS, restrictive allograft syndrome; sHR, subdistribution hazard ratio

Because PVR may better reflect pulmonary vascular burden than mPAP alone, we evaluated PVR as an additional predictor of waitlist mortality. In adjusted Fine–Gray analysis, each 1-WU increase in PVR was associated with higher waitlist mortality (sHR 1.13, 95% CI 1.04-1.22; *p*=0.002). When modeled categorically, PVR ≥3 WU was associated with increased waitlist mortality compared with PVR <2 WU (sHR 1.43, 95% CI 1.08-1.88; *p*=0.011), whereas PVR 2 to <3 WU was not significant (Supplementary Table 6). In a hemodynamic phenotype sensitivity analysis using no PH as the reference group, combined pre- and post-capillary PH was associated with higher waitlist mortality (sHR 1.59, 95% CI 1.04-2.45; *p*=0.034), whereas pre-capillary PH showed a nonsignificant trend (sHR 1.29, 95% CI 0.97-1.71; *p*=0.076) and isolated post-capillary PH was not significantly associated with waitlist mortality (sHR 1.18, 95% CI 0.77-1.80; *p*=0.458; Supplementary Table 7).

Patients with PH were less likely to undergo retransplantation than patients without PH (613/813 [75.4%] vs 461/574 [80.3%], *p*=0.037). Among retransplanted patients, median time from relisting to retransplantation was similar by PH status (0.11 years [IQR 0.04-0.31] vs 0.11 years [IQR 0.05-0.31], *p*=0.368). In unadjusted competing-risk analyses, cumulative incidence differed by PH status for both waitlist mortality and retransplantation (Gray’s test *p*=0.037 and *p*=0.026, respectively; Supplementary Table 5 and Supplementary Figure 3).

Next, to delineate whether these findings were relevant in the new allocation system, we performed a sensitivity analysis restricted to patients in the CAS era. In this cohort, out of 254 patients, 225 patients with complete data were included. Baseline characteristics were largely similar between patients with and without PH except for higher Hispanic representation in the PH group (Supplementary Table 1). PH was not significantly associated with waitlist mortality. Although the point estimate suggested a higher risk, the analysis was limited by the smaller sample size and number of events in the CAS-era cohort (Supplementary Table 2). In this model, female sex was nominally associated with higher waitlist mortality (sHR 3.08, 95% CI 1.02-9.29), with a wide confidence interval consistent with the limited number of events. In the unadjusted Kaplan–Meier analysis, survival from listing did not differ by PH status (Supplementary Figure 2).

### Cause-specific sensitivity analysis

In a sensitivity analysis using cause-specific Cox proportional hazards regression with transplantation treated as a censoring event, PH was not significantly associated with waitlist.

mortality (HR 1.11, 95% CI 0.97-1.27, *p* = 0.136) (Table 4). Other factors independently associated with mortality in this model included pre-listing ECMO support, diabetes, lower body mass index, and RAS phenotype.

**Table 4:** Cause-Specific Cox Proportional Hazards Model for Waitlist Mortality Among CLAD Retransplant Candidates (Sensitivity Analysis)

**Variable**	**Hazard Ratio (HR)**	**95% CI**	***p*-value**
Pulmonary hypertension	1.11	0.97-1.27	0.136
Age (year)	1.00	1.00-1.01	0.741
Body mass index (kg/m²)	0.98	0.96-0.99	0.004
BOS phenotype (vs RAS)	0.75	0.64-0.88	<0.001
Creatinine (mg/dL)	0.86	0.74-1.00	0.054
Diabetes mellitus	1.20	1.05-1.36	0.006
ECMO support at listing	2.43	1.43-4.14	0.001

Cause-specific Cox proportional hazards regression was performed treating lung transplantation as a censoring event. Hazard ratios represent the instantaneous risk of death among patients remaining on the waitlist. BOS, bronchiolitis obliterans syndrome; ECMO, extracorporeal membrane oxygenation; HR, hazard ratio; RAS, restrictive allograft syndrome

### Subgroup analysis by CLAD phenotype

In phenotype-stratified analyses (Supplementary Table 3)**,** PH demonstrated a marginal association with waitlist mortality in the BOS subgroup (HR 1.14, 95% CI 0.98-1.34, *p* = 0.097) but was not associated with mortality in the RAS subgroup (HR 1.06, 95% CI 0.78-1.44, *p* = 0.708). In patients with RAS, mortality was strongly associated with ECMO support (HR 4.87, 95% CI 2.06-11.52, *p* < 0.001) and diabetes (HR 1.66, 95% CI 1.24-2.22, *p* = 0.001).

### Descriptive survival analysis

**Figure 2:**
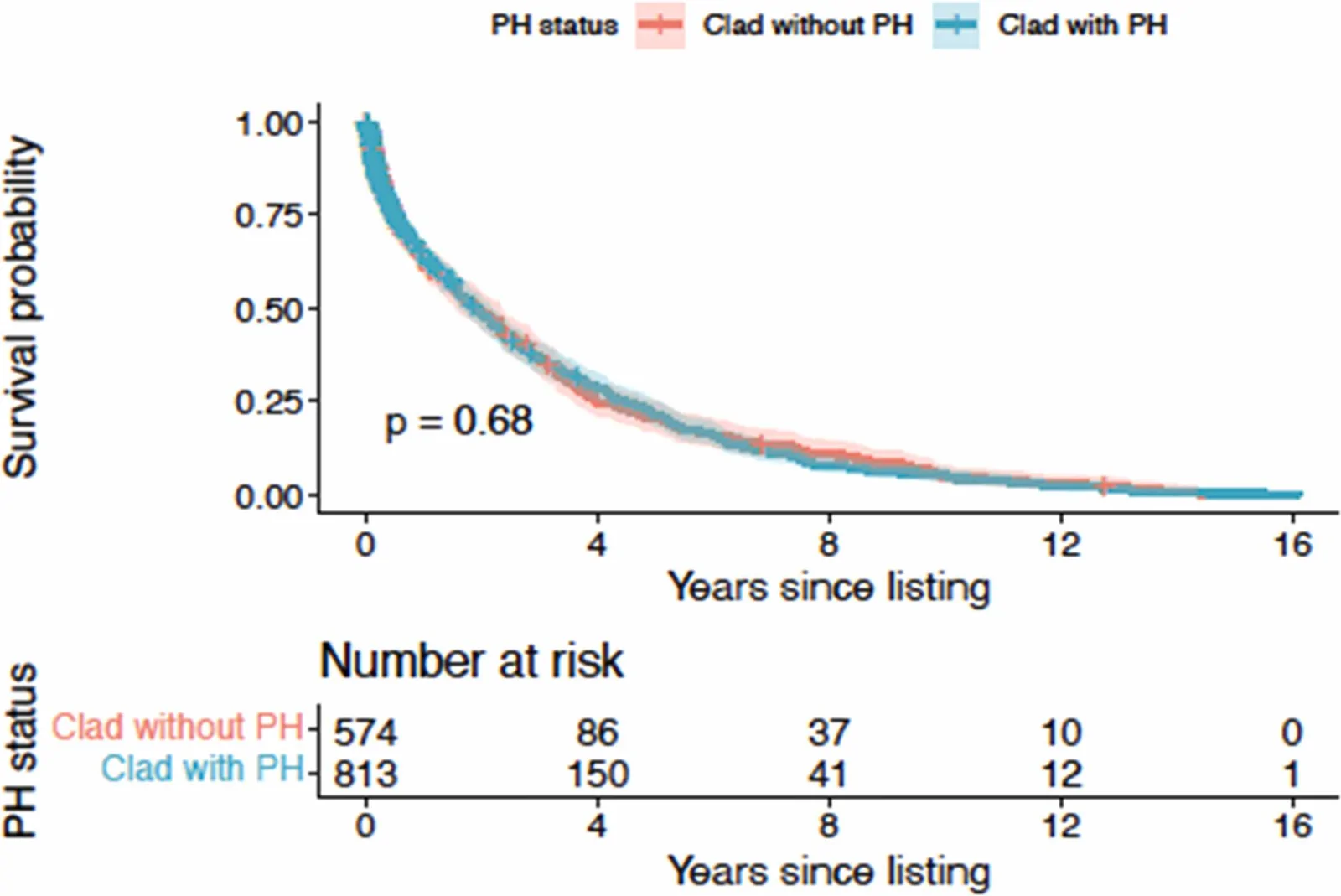
Kaplan–Meier survival from listing among CLAD retransplant candidates with and without pulmonary hypertension. Kaplan–Meier survival curves comparing CLAD retransplant candidates with and without pulmonary hypertension at listing. Survival did not differ significantly between groups (log-rank *p* = 0.68). Because transplantation represents a competing event for waitlist mortality, Kaplan–Meier estimates are presented for descriptive purposes. CLAD, chronic lung allograft dysfunction

In an unadjusted Kaplan–Meier analysis, survival from the time of listing did not differ significantly between patients with and without PH (log-rank *p* = 0.68) (Figure 2). Median survival and restricted mean survival time at 5 years were similar between groups. As these analyses do not account for the competing risk of transplantation, they were considered descriptive. To complement the primary Fine–Gray competing-risk analysis, cumulative incidence curves by PH status are shown in Supplementary Figure 3.

### Post-transplant outcomes

Among the 1074 patients who underwent retransplantation, early postoperative complications were broadly similar between those with and without PH. There were no significant differences in airway dehiscence, need for dialysis, transfusion requirements, tracheostomy, ECMO support within 72 hours, or length of hospital stay (Table 5). However, patients with PH were more likely to remain intubated at 72 hours after retransplantation than those without PH (46.3% vs 36.3%, *p* = 0.027). PH was not associated with 30-day post-transplant mortality (OR 0.98, *p* = 0.943) or 1-year mortality (OR 1.05, *p* = 0.781) (Supplementary Table 4). Diabetes and pre-transplant ECMO support were strong predictors of early post-transplant mortality, whereas BOS phenotype was associated with lower 1-year mortality than RAS.

**Table 5:** Early Post-transplant Outcomes Among CLAD Patients Undergoing Lung Retransplantation

**Outcome**	**CLAD without PH (n=461)**	**CLAD with PH (n=613)**	***p*-value**
Airway dehiscence	8 (1.8%)	13 (2.1%)	0.528
Dialysis	61 (14.8%)	99 (16.3%)	0.110
Transfusion requirement	60 (13.2%)	87 (14.3%)	0.788
Prolonged mechanical ventilation	53 (11.5%)	93 (15.2%)	0.099
ECMO support at 72 h	42 (13.8%)	57 (14.7%)	0.932
Intubation at 72 h	110 (36.3%)	180 (46.3%)	0.027
Tracheostomy	53 (11.7%)	76 (12.5%)	0.707
Length of stay (days)	30.33±35.44	35.32±50.96	0.076

Denominators vary by outcome because of missing registry data. Percentages were calculated using the number of patients with non-missing data for each outcome

## Discussion

Using a large national registry cohort of adults listed for lung retransplantation for CLAD, this study found that PH was present in more than half of repeat candidates at the time of listing. PH was independently associated with increased waitlist mortality when transplantation was appropriately treated as a competing event (adjusted sHR 1.37, 95% CI 1.08-1.74; *p*=0.009). By contrast, PH was not associated with increased 30-day or 1-year mortality after retransplantation. Patients with PH were, however, more likely to remain intubated at 72 hours after transplant, suggesting greater early postoperative vulnerability despite similar short-term survival.

Notably, the prevalence of PH in this cohort was higher than that reported in earlier registry-based analyses of patients relisted for BOS.^8^ Several factors likely explain this difference. First, our study used contemporary hemodynamic criteria that define PH as mPAP greater than 20 mmHg,^10^ rather than the historical threshold of 25 mmHg or greater.^11^ Second, our analysis relied on RHC data recorded at listing across a national registry cohort from 2005 to 2025, during which recipient complexity and comorbidity burden may differ from earlier cohorts. Third and importantly, we included both BOS and RAS and differentiated them rather than BOS alone. Taken together, these differences likely account for the higher observed prevalence and support the view that PH is a common feature of advanced CLAD in present-day practice.

A central finding of the current study is that PH was associated with increased waitlist mortality in competing-risk (Fine–Gray) analysis, even after adjustment for other relevant covariates. However, this association was not observed in the cause-specific Cox model. This contrast deserves explicit interpretation, because the 2 approaches answer different clinical questions. The cause-specific Cox model estimates the instantaneous risk of death among patients still on the waitlist; in this model, PH was not significantly associated with mortality (HR 1.11, *p*=0.136), suggesting that PH patients on the waitlist do not die at a meaningfully higher rate per unit time than non-PH patients. The Fine–Gray model, in contrast, estimates the cumulative probability of dying before transplantation, treating transplantation as a competing event that prevents waitlist death; in this model, PH was associated with significantly increased waitlist mortality (sHR 1.37, *p*=0.009). Additional retransplantation analyses help contextualize this discrepancy. Patients with PH were less likely to undergo retransplantation overall, although median time to retransplantation among those retransplanted was similar by PH status. Therefore, the Fine–Gray finding should not be interpreted as definitive evidence that PH directly accelerates instantaneous waitlist death. Rather, PH appears to identify a clinically vulnerable subgroup with higher cumulative incidence of death before retransplantation and lower cumulative retransplantation overall. The absence of a statistically significant association in the CAS-era subgroup should be interpreted cautiously. Because CAS was implemented only in March 2023, this subgroup had fewer patients, shorter follow-up, and fewer waitlist mortality events, which likely limited statistical power and precision. However, a true allocation-era effect cannot be excluded, as CAS incorporates a broader set of medical urgency and expected post-transplant survival variables than LAS and may alter transplant access for candidates with PH. Longer follow-up in larger CAS-era cohorts will be needed to determine whether CAS modifies the association between PH and waitlist outcomes.

The association between PH and waitlist mortality may also be influenced by survivorship bias and cumulative allograft exposure. Patients who survive longer after their first transplant may have more time to develop pulmonary vascular consequences of chronic allograft injury, parenchymal remodeling, hypoxic vasoconstriction, and progressive physiologic frailty. Although patients with PH were only slightly older at relisting and age was included in multivariable models, age may not fully capture time since first transplant, CLAD duration, or chronic respiratory failure burden. Therefore, PH may function partly as a marker of more advanced CLAD and cumulative graft injury rather than as an isolated vascular risk factor.

The inverse association between BMI and waitlist mortality in the cause-specific Cox model may reflect the adverse prognostic significance of low body mass in advanced CLAD. In retransplant candidates, lower BMI may serve as a surrogate for cachexia, sarcopenia, nutritional depletion, and reduced physiologic reserve related to severe chronic graft dysfunction, rather than indicating that higher BMI is directly protective. The directionally similar association in the Fine–Gray model, although not statistically significant, supports cautious interpretation. However, because registry data did not include more granular measures of sarcopenia, frailty, or longitudinal weight loss, the mechanisms underlying this association could not be directly assessed.

Beyond the binary PH definition, when mPAP was examined as a continuous variable, the risk of waitlist mortality increased progressively with higher mPAPs, without evidence of a threshold effect. This finding suggests that the relationship between pulmonary vascular burden and pre-transplant risk in CLAD is better understood as a continuum rather than a binary state. Even modest elevations in pulmonary pressure may therefore carry prognostic significance in retransplant candidates.

The hemodynamic data also provide insight into the nature of PH in this population. Patients with CLAD demonstrated evidence of mixed hemodynamic phenotypes rather than a purely pre-capillary process. Among patients with complete data, pre-capillary PH was somewhat more frequent in RAS than in BOS, although the difference was not statistically significant, whereas post-capillary PH occurred at a similar frequency in both phenotypes. These findings suggest that pulmonary vascular disease in CLAD likely reflects overlapping contributions from parenchymal distortion, hypoxic vasoconstriction, pulmonary vascular remodeling,^6^, 12. and, in some patients, elevated left-sided filling pressures.^8^ The modest excess of pre-capillary PH in RAS is biologically plausible given the greater parenchymal and fibrotic burden typically seen in that phenotype. Restrictive CLAD is characterized by pleuroparenchymal remodeling, reduced lung compliance, and architectural distortion, which may contribute to hypoxic vasoconstriction, microvascular loss, and increased PVR. However, because this difference was not statistically significant, this finding should be interpreted as hypothesis-generating and highlights hemodynamic heterogeneity across both major forms of CLAD. The hemodynamic phenotype sensitivity analysis further supports this heterogeneity. Combined pre- and post-capillary PH showed the strongest association with waitlist mortality, whereas pre-capillary PH showed a nonsignificant trend and isolated post-capillary PH was not significantly associated with waitlist mortality.

Post-capillary PH was present in a substantial proportion of patients. Candidates for retransplantation often have concomitant systemic and cardiac comorbidities, and long-term exposure to calcineurin inhibitors and corticosteroids may contribute to hypertension, renal dysfunction, and myocardial remodeling.^14^, ^15^, ^17^, ^16^ Additional mechanisms may include post capillary processes related to immune-mediated injury in chronic rejection, including potential involvement of the pulmonary venous circulation. Earlier registry analyses of BOS also identified elevated wedge pressure in a sizeable proportion of patients with PH,^8^ and our findings extend those observations to a broader CLAD population. Overall, these data support the view that PH in CLAD is not a single hemodynamic entity, but rather a composite manifestation of advanced graft dysfunction and competing cardiopulmonary influences.

Despite its association with waitlist mortality, PH was not independently associated with early or 1-year mortality following retransplantation. One explanation is that PH in this setting may largely reflect the hemodynamic consequences of the failing allograft and severe chronic lung injury, both of which may be alleviated after replacement of the graft. Another possibility is that patients with the most severe cardiopulmonary compromise do not survive to retransplantation, such that those who ultimately undergo surgery represent a more selected subgroup with sufficient reserve to tolerate the operation. In addition, the lack of association with post-transplant mortality may reflect the relatively short follow-up period. These considerations may explain why PH identifies important pre-transplant risk but does not translate into excess short-term mortality after retransplantation.

Nevertheless, the greater likelihood of remaining intubated at 72 hours after retransplantation suggests that PH may still carry peri- and postoperative consequences. This finding may reflect reduced right ventricular reserve, delayed postoperative hemodynamic adaptation, greater pulmonary vascular burden, impaired early gas exchange, or greater overall physiological frailty related to advanced CLAD. It may also reflect subclinical primary graft dysfunction (PGD). Our dataset did not include adjudicated PGD outcomes, limiting definitive mechanistic conclusions, but the result suggests PH may contribute to a more complex immediate postoperative course even when short-term survival is preserved. Diamond et al. established PH as an independent risk factor for grade 3 PGD in primary lung transplant recipients in a multi-institutional study, so further exploration in retransplant recipients with more granular data would assist in delineating the differences found in our study cohorts.^18^

The biological basis for PH in CLAD is likely multifactorial. Although CLAD has historically been framed as an airway-dominant disorder, histopathological studies of explanted CLAD lungs have demonstrated substantial vascular involvement, including arteriopathy, venopathy, endothelial inflammation, intimal hyperplasia, and extracellular matrix deposition.^6^, 12. Chronic hypoxemia from impaired gas exchange may promote sustained vasoconstriction and vascular smooth muscle proliferation,^19^ while persistent alloimmune injury and inflammatory signaling may further drive endothelial dysfunction and remodeling.^4^, ^20^ In addition, microvascular rarefaction and capillary loss may contribute to increased pulmonary vascular resistance in advanced disease.^21^, ^22^ Taken together, these observations support the concept that pulmonary vascular remodeling is an integral component of chronic graft failure rather than merely a secondary bystander process. The PVR sensitivity analyses support this interpretation. Elevated PVR was associated with waitlist mortality when modeled continuously, and PVR ≥3 WU identified patients with higher cumulative incidence of waitlist death. In addition, the strongest hemodynamic subtype signal was observed among patients with combined pre- and post-capillary PH. These findings suggest that pulmonary vascular burden may provide prognostic information beyond mPAP alone and support the biological relevance of pulmonary vascular remodeling in CLAD retransplant candidates.

Beyond pulmonary vascular biology, our multivariable model also identified an unexpected demographic finding. Hispanic ethnicity was independently associated with lower waitlist mortality (sHR 0.62, 95% CI 0.40-0.97; *p*=0.038). This is consistent with the "Hispanic paradox," in which Hispanic populations demonstrate lower all-cause mortality despite a less favorable socioeconomic profile, and which has been reported in kidney, liver, and lung transplantation.^23^, ^24^, ^25^ However, Hispanic candidates remain less likely than White candidates to receive a lung transplant after listing,^26^ raising the possibility that selection effects or differential transplant rates contribute to this observation. Hispanic ethnicity was included in the adjusted model because it had sufficient sample size and prior relevance to transplant access and outcomes; however, this finding was not a primary study objective. Because we could not evaluate nativity, primary language, or socioeconomic status, this finding is hypothesis-generating.

Our findings also have potential clinical implications. Identification of PH during retransplant evaluation may help refine risk assessment beyond existing allocation metrics and could prompt more comprehensive hemodynamic assessment and earlier referral for advanced transplant evaluation. Whether targeted hemodynamic optimization or PH-directed strategies could improve symptoms, exercise tolerance, or waitlist outcomes in selected patients with CLAD remains uncertain and deserves further study. At a minimum, these data support careful attention to pulmonary vascular status as part of the evaluation of retransplant candidates.

This study has several limitations. Its retrospective design introduces the possibility of residual confounding despite multivariable adjustment. Registry-based analyses are limited by the granularity and completeness of recorded data, including incomplete hemodynamic variables for some patients and a lack of detailed physiological, imaging, and treatment information. In addition, missing hemodynamic data resulted in exclusion of 274 patients (16.0%) from the primary analytic cohort, which may have influenced cohort composition and introduced selection bias if patients without complete hemodynamic data differed systematically from those included. We were unable to directly assess mechanisms of PH, longitudinal hemodynamic trajectories, or the use and impact of PH-directed therapies. We also lacked reliable data on CLAD onset and time from first transplantation to relisting, limiting our ability to assess cumulative allograft exposure or survivorship bias. Postoperative outcomes available in the registry do not fully capture complications such as PGD or detailed measures of right ventricular performance, or broader airway complications beyond registry-reported airway dehiscence. Nonetheless, the study draws on a large national registry cohort with RHC data and provides clinically relevant insights into the prevalence and prognostic importance of PH among CLAD retransplant candidates.

In conclusion, PH is common in adults listed for lung retransplantation for CLAD and is independently associated with increased waitlist mortality, but not with early post-transplant mortality. The discordance between competing-risk and cause-specific analyses suggests that PH identifies a clinically vulnerable subgroup with higher cumulative incidence of death before retransplantation and lower overall retransplantation. These findings support PH as an important marker of pre-transplant risk in CLAD retransplant candidates. PH in this setting appears hemodynamically heterogeneous, with both pre-capillary and post-capillary forms across CLAD phenotypes. These findings support routine hemodynamic assessment during retransplant evaluation and prospective evaluation of whether allocation criteria adequately reflect the risk conferred by PH in the CAS era.

## Disclosures

The authors declare that they have no competing interests related to this manuscript.

## Funding

The study was supported by 10.13039/100000002NIH grant R01HL161620 to N.S.S.

## CRediT authorship contribution statement

P.C., N.S.S. developed the concept; P.C., N.S.S., K.W., A.S.P. analyzed the data; P.C., N.S.S., K.W., R.F, E.S., G.Li., G.L.,D.H, A.S.P., D.D.M, J.S., M.H.S., H.M.P, C.V., Y.G., M.B performed a critical review of the manuscript and revised accordingly.

## Declaration of Generative AI and AI-assisted technologies in the writing process

During the preparation of this work, the authors used Claude AI to check grammar and spelling. The authors have reviewed and confirmed the validity of the text and take full responsibility for the content of the publication.

## Declaration of Competing Interest

The authors declare that they have no known competing financial interests or personal relationships that could have appeared to influence the work reported in this paper.

## Data Availability

Data are available from the Scientific Registry of Transplant Recipients upon application.

## Footnotes

Appendix A Supplementary data associated with this article can be found in the online version at doi:10.1016/j.jhlto.2026.100651.
