# Supplementary material for: National prevalence and clinical impact of pulmonary hypertension in adults listed for lung retransplantation for chronic lung allograft dysfunction
Source: JHLT Open. 2026 Aug 3;14:100651. doi: 10.1016/j.jhlto.2026.100651 (PMC13503125; doi:10.1016/j.jhlto.2026.100651)
Supplement: Supplementary file 1 [file mmc1.docx]

**Supplementary Table 1. Baseline Characteristics of CAS-Era Cohort Stratified by Pulmonary Hypertension Status**

| **Characteristic** | **CLAD without PH (n = 115)** | **CLAD with PH (n = 139)** | **p-value** |
| --- | --- | --- | --- |
| **Demographics** |  |  |  |
| Age at listing, years | 47.2 ± 11.9 | 46.3 ± 13.3 | 0.596 |
| Female sex, n (%) | 53 (46.1) | 65 (46.8) | 1.000 |
| Hispanic ethnicity, n (%) | 10 (8.7) | 26 (18.7) | 0.036 |
| **Clinical characteristics** |  |  |  |
| Body mass index, kg/m² | 23.8 ± 15.0 | 25.4 ± 11.2 | 0.337 |
| Current or prior smoking, n (%) | 27 (23.5) | 39 (28.1) | 0.494 |
| Diabetes, n (%) | 29 (25.2) | 41 (29.5) | 0.624 |
| Creatinine, mg/dL | 1.07 ± 0.55 | 1.14 ± 0.83 | 0.430 |
| Oxygen requirement, L/min | 10.3 ± 10.8 | 6.7 ± 8.6 | 0.095 |
| **Disease characteristics** |  |  |  |
| CLAD phenotype (BOS), n (%) | 88 (76.5) | 109 (78.4) | 0.834 |

Values are presented as mean ± SD or n (%).

Abbreviations: BMI = body mass index, BOS = bronchiolitis obliterans syndrome, CLAD = chronic lung allograft dysfunction, PH = pulmonary hypertension

**Supplementary Table 2. Multivariable Fine–Gray Competing-Risk Regression for Waitlist Mortality in the CAS-Era Cohort**

| **Variable** | **Subdistribution Hazard Ratio (sHR)** | **95% CI** | **p-value** |
| --- | --- | --- | --- |
| Pulmonary hypertension | 2.79 | 0.85–9.16 | 0.091 |
| Age (year) | 1.00 | 0.95–1.05 | 0.929 |
| Female sex | 3.08 | 1.02–9.29 | 0.046 |
| Body mass index (kg/m²) | 0.95 | 0.85–1.07 | 0.436 |
| Hispanic ethnicity | 1.03 | 0.27–3.97 | 0.963 |
| BOS phenotype (vs RAS) | 0.82 | 0.26–2.54 | 0.728 |
| Creatinine (mg/dL) | 1.53 | 0.92–2.54 | 0.104 |
| Diabetes mellitus | 0.70 | 0.27–1.81 | 0.466 |
| ECMO support at listing | 1.94 | 0.24–15.45 | 0.529 |

Fine–Gray competing-risk regression was used to evaluate waitlist mortality with lung transplantation treated as a competing event. Models were adjusted for clinically relevant covariates selected a priori.

**Abbreviations**

CLAD = chronic lung allograft dysfunction
BOS = bronchiolitis obliterans syndrome
RAS = restrictive allograft syndrome
ECMO = extracorporeal membrane oxygenation
sHR = subdistribution hazard ratio

**Supplementary Table 3. Cause-Specific Cox Models for Waitlist Mortality Stratified by CLAD Phenotype**

| **Variable** | **BOS HR (95% CI)** | **p-value** | **RAS HR (95% CI)** | **p-value** |
| --- | --- | --- | --- | --- |
| Pulmonary hypertension | 1.14 (0.98–1.34) | 0.097 | 1.06 (0.78–1.44) | 0.708 |
| Age | 1.00 (0.99–1.01) | 0.079 | 1.01 (0.99–1.02) | 0.370 |
| Female sex | 0.83 (0.71–0.96) | 0.015 | 1.16 (0.86–1.57) | 0.342 |
| Body mass index | 0.97 (0.95–0.99) | 0.002 | 0.99 (0.96–1.03) | 0.648 |
| Creatinine | 0.83 (0.70–1.00) | 0.046 | 0.87 (0.63–1.20) | 0.396 |
| Diabetes | 1.07 (0.93–1.24) | 0.350 | 1.66 (1.24–2.22) | 0.001 |
| ECMO at listing | 1.59 (0.79–3.22) | 0.194 | 4.87 (2.06–11.52) | <0.001 |

Cause-specific Cox proportional hazards models stratified by CLAD phenotype. Hazard ratios represent the instantaneous risk of death among patients remaining on the waitlist.

**Supplementary Table 4**. **Multivariable logistic regression models for post-transplant mortality (30-day and 1-year)**

| **Variable** | **30-day mortality OR (95% CI)** | **p-value** | **1-year mortality OR (95% CI)** | **p-value** |
| --- | --- | --- | --- | --- |
| Pulmonary Hypertension | 0.98 (0.56–1.71) | 0.943 | 1.05 (0.76–1.45) | 0.781 |
| Age | 1.02 (1.00–1.04) | 0.085 | 1.01 (1.00–1.03) | 0.046 |
| Female sex | 0.88 (0.50–1.53) | 0.646 | 1.00 (0.72–1.37) | 0.986 |
| BMI | 1.00 (0.96–1.05) | 0.991 | 0.98 (0.94–1.01) | 0.203 |
| Hispanic ethnicity | 0.60 (0.21–1.73) | 0.346 | 0.95 (0.57–1.60) | 0.852 |
| BOS phenotype | 0.58 (0.32–1.03) | 0.063 | 0.66 (0.46–0.94) | 0.023 |
| Creatinine | 0.99 (0.66–1.49) | 0.968 | 0.99 (0.78–1.26) | 0.939 |
| Diabetes | 2.66 (1.49–4.77) | 0.001 | 1.50 (1.09–2.06) | 0.013 |
| ECMO support | 4.62 (1.57–13.63) | 0.006 | 2.28 (0.93–5.56) | 0.071 |

Models include all listed covariates. These analyses were exploratory and limited to patients who underwent lung re-transplantation.

**Supplementary Table 5. Retransplantation by pulmonary hypertension status**

| **PH status** | **N** | **Retransplanted, n (%)** | **Time from relisting to retransplantation among retransplanted, median years (IQR)** |
| --- | --- | --- | --- |
| **CLAD without PH** | **574** | **461 (80.3)** | **0.11 (0.05–0.31)** |
| **CLAD with PH** | **813** | **613 (75.4)** | **0.11 (0.04–0.31)** |

P=0.0365 for comparison of retransplantation proportion by PH status; p=0.3678 for comparison of time from relisting to retransplantation among retransplanted patients.

**Supplementary Table 6. Fine-Gray competing-risk models evaluating pulmonary vascular resistance and waitlist mortality**

| **Model** | **PVR variable** | **sHR** | **95% CI** | **p value** |
| --- | --- | --- | --- | --- |
| **PVR as continuous predictor** | **Per 1-WU increase** | **1.13** | **1.04–1.22** | **0.002** |
| **PVR category** | **2 to <3 WU vs <2 WU** | **0.94** | **0.71–1.24** | **0.644** |
| **PVR category** | **≥3 WU vs <2 WU** | **1.43** | **1.08–1.88** | **0.011** |

Fine–Gray competing-risk regression was used to evaluate waitlist mortality, with retransplantation treated as a competing event. Models were adjusted for age, sex, body mass index, ethnicity, CLAD phenotype, serum creatinine, diabetes, and ECMO support at listing. For the categorical model, PVR <2 WU was used as the reference group. CLAD = chronic lung allograft dysfunction; PVR = pulmonary vascular resistance; sHR = subdistribution hazard ratio; WU = Wood units.

**Supplementary Table 7. Fine–Gray competing-risk model for waitlist mortality by hemodynamic PH phenotype**

| **Variable** | **sHR** | **95% CI** | **p value** |
| --- | --- | --- | --- |
| Pre-capillary PH vs no PH | 1.29 | 0.97–1.71 | 0.076 |
| Isolated post-capillary PH vs no PH | 1.18 | 0.77–1.80 | 0.458 |
| Combined pre/post-capillary PH vs no PH | 1.59 | 1.04–2.45 | 0.034 |
| Age | 0.99 | 0.98–1.00 | 0.287 |
| Female sex | 0.90 | 0.70–1.15 | 0.388 |
| BMI | 0.98 | 0.95–1.01 | 0.169 |
| Hispanic ethnicity | 0.60 | 0.37–0.98 | 0.040 |
| BOS phenotype | 0.97 | 0.72–1.32 | 0.858 |
| Serum creatinine | 0.88 | 0.70–1.12 | 0.298 |
| Diabetes | 0.86 | 0.67–1.10 | 0.238 |
| ECMO support at listing | 0.47 | 0.11–2.01 | 0.308 |

Fine–Gray competing-risk regression was used to evaluate waitlist mortality, with retransplantation treated as a competing event. No PH was used as the reference group for hemodynamic phenotype. The model was adjusted for age, sex, body mass index, ethnicity, CLAD phenotype, serum creatinine, diabetes, and ECMO support at listing. BMI = body mass index; BOS = bronchiolitis obliterans syndrome; CLAD = chronic lung allograft dysfunction; ECMO = extracorporeal membrane oxygenation; PH = pulmonary hypertension; sHR = subdistribution hazard ratio.

**Supplementary Table 8. Pulmonary Hypertension Prevalence and Hemodynamic Characteristics Across Three CLAD Phenotype Groups**

| **Characteristic** | **BOS/obstructive** | **Pure restrictive** | **Mixed obstructive-restrictive** | **p-value** |
| --- | --- | --- | --- | --- |
| Patients, n | 1,371 | 74 | 269 | - |
| Patients with hemodynamic data sufficient to determine PH status, n | 1,135 | 69 | 236 | - |
| PH, n (%) | 662 (58.3) | 41 (59.4) | 134 (56.8) | 0.886 |
| **Hemodynamic phenotype** |  |  |  | 0.796 |
| Pre-capillary PH, n (%) | 320 (28.2) | 22 (31.9) | 74 (31.4) |  |
| Isolated post-capillary PH, n (%) | 112 (9.9) | 7 (10.1) | 25 (10.6) |  |
| Combined pre- and post-capillary PH, n (%) | 79 (7.0) | 4 (5.8) | 14 (5.9) |  |
| PH not further classifiable, n (%) | 151 (13.3) | 8 (11.6) | 21 (8.9) |  |
| **Hemodynamic findings** |  |  |  |  |
| mPAP, mmHg | 22.5 ± 6.9 | 23.0 ± 8.6 | 22.4 ± 7.2 | 0.887 |
| PCWP, mmHg | 11.1 ± 5.3 | 10.5 ± 6.8 | 10.5 ± 5.6 | 0.061 |
| PVR, Wood units | 2.3 ± 1.3 | 2.5 ± 1.4 | 2.5 ± 1.6 | 0.084 |
| CO, L/min | 5.3 ± 1.7 | 5.3 ± 1.3 | 5.3 ± 1.6 | 0.922 |

Values are presented as n (%) or mean ± SD. Classifiable PH status refers to patients with hemodynamic data sufficient to determine PH status. PH was defined as mPAP >20 mmHg. Hemodynamic phenotypes were mutually exclusive. Mixed obstructive-restrictive phenotype refers to registry coding for obliterative bronchiolitis–restrictive. P-values for continuous variables were calculated using Kruskal–Wallis tests; categorical variables were compared using chi-square or Fisher’s exact tests, as appropriate.

**Supplementary Table 9. Fine–Gray Sensitivity Model Using Three-Group CLAD Phenotype Classification**

| **Variable** | **sHR** | **95% CI** | **p-value** |
| --- | --- | --- | --- |
| PH | 1.37 | 1.08–1.73 | 0.010 |
| Pure restrictive phenotype | 0.63 | 0.32–1.24 | 0.181 |
| Mixed obstructive-restrictive phenotype | 1.07 | 0.79–1.44 | 0.676 |
| Age at listing | 0.99 | 0.99–1.00 | 0.245 |
| Female sex | 0.87 | 0.70–1.10 | 0.251 |
| Body mass index | 0.99 | 0.96–1.01 | 0.222 |
| Hispanic ethnicity | 0.63 | 0.40–0.99 | 0.043 |
| Creatinine | 0.93 | 0.77–1.13 | 0.469 |
| Diabetes | 0.88 | 0.70–1.10 | 0.265 |
| ECMO support at listing | 0.35 | 0.08–1.45 | 0.147 |

Fine–Gray competing-risk regression was used to evaluate waitlist mortality, with retransplantation treated as a competing event. BOS/obstructive phenotype was used as the reference group for CLAD phenotype. Abbreviations: CLAD = chronic lung allograft dysfunction; ECMO = extracorporeal membrane oxygenation; PH = pulmonary hypertension; sHR = subdistribution hazard ratio.

**Supplementary Table 10. Hemodynamic Phenotype Distribution Overall and by CLAD Phenotype**

|  | **Overall (n = 1,440)** | **BOS/obstructive (n = 1,135)** | **RAS/restrictive (n = 305)** | **P-value** |
| --- | --- | --- | --- | --- |
| PH, n (%) | 837 (58.1) | 662 (58.3) | 175 (57.4) | 0.766 |
| **Hemodynamic phenotype** |  |  |  | 0.378 |
| Pre-capillary PH, n (%) | 416 (28.9) | 320 (28.2) | 96 (31.5) |  |
| Isolated post-capillary PH, n (%) | 144 (10.0) | 112 (9.9) | 32 (10.5) |  |
| Combined pre- and post-capillary PH, n (%) | 97 (6.7) | 79 (7.0) | 18 (5.9) |  |
| PH not further classifiable due to missing PCWP/PVR, n (%) | 180 (12.5) | 151 (13.3) | 29 (9.5) |  |

Percentages are calculated within each column denominator. Hemodynamic phenotype categories are mutually exclusive and sum to the overall PH prevalence.

**Supplementary Table 11. Missing Data for Variables Included in Descriptive Tables and Analyses**

| **Variable** | **Missing, n (%)** |
| --- | --- |
| Age at listing | 0 (0.0) |
| Sex | 0 (0.0) |
| Body mass index | 2 (0.14) |
| Race/ethnicity | 0 (0.0) |
| Ethnicity | 0 (0.0) |
| CLAD phenotype | 0 (0.0) |
| Diabetes mellitus | 0 (0.0) |
| Current or prior smoking | 0 (0.0) |
| Serum creatinine | 0 (0.0) |
| Serum albumin | 1,046 (72.6) |
| Oxygen requirement at listing | 273 (19) |
| Oxygen supplementation at listing | 273 (19) |
| LAS at listing | 227 (15.76) |
| LAS at matching | 258 (17.92) |
| ECMO support at listing | 0 (0.0) |
| Systolic pulmonary artery pressure | 1 (0.07) |
| Diastolic pulmonary artery pressure | 3 (0.21) |
| Mean pulmonary artery pressure | 0 (0.0) |
| Pulmonary capillary wedge pressure | 27 (1.88) |
| Cardiac output | 39 (2.71) |
| Pulmonary vascular resistance | 67 (4.65) |
| Airway dehiscence | 26 (2.42) |
| Dialysis | 26 (2.42) |
| Transfusion requirement | 37 (3.45) |
| Prolonged mechanical ventilation | 0 (0.0) |
| Tracheostomy | 18 (1.68) |
| ECMO support at 72 hours | 401 (37.34) |
| Intubation at 72 hours | 401 (37.34) |
| Length of stay | 36 (3.35) |

Missing data are reported for variables included in the descriptive tables and analyses. Percentages for baseline and hemodynamic variables were calculated among the primary analytic cohort with PH status determined (n=1,440). Percentages for post-transplant outcomes were calculated among patients who underwent retransplantation (n=1,074). PH status could not be determined in 274 of 1,714 eligible patients (16.0%) because of missing hemodynamic data; these patients were excluded from the primary analytic cohort.

**Supplementary Figure 1: Study Flow Diagram**

**SRTR May 2005-Sept 2025**

**N= 1714 CLAD patients listed for retransplants**

**Analytic Cohort n=1440**

**Excluded= 274**

**Insufficient hemodynamic data**

**Classified by mPAP>20**

**No PH**

**(n=603, 41.9%)**

**PH**

**(n=837, 58.1%)**

**Primary outcome**

**waitlist mortality**

**n=1440**

**Post-transplant**

**N=1074**

**Cas-era subgroup**

**N=225**

**Supplementary Figure 2.** Kaplan–Meier survival from listing among CLAD retransplant candidates in the CAS era with and without pulmonary hypertension. Kaplan–Meier survival curves comparing CLAD retransplant candidates in the CAS era with and without pulmonary hypertension at listing. Survival did not differ significantly between groups (log-rank p = 0.16). Because transplantation represents a competing event for waitlist mortality, Kaplan–Meier estimates are presented for descriptive purposes.

**Supplementary Figure 3. Cumulative incidence of waitlist mortality and retransplantation by pulmonary hypertension status.**

Cumulative incidence curves are shown for waitlist mortality and retransplantation after listing for lung retransplantation, stratified by pulmonary hypertension (PH) status. In the figure, **PH=0: Death** represents cumulative waitlist death among patients without PH; **PH=1: Death** represents cumulative waitlist death among patients with PH; **PH=0: Transplant** represents cumulative retransplantation among patients without PH; and **PH=1: Transplant** represents cumulative retransplantation among patients with PH. Because retransplantation precludes waitlist death, retransplantation was treated as a competing event for waitlist mortality; conversely, waitlist death was treated as a competing event for retransplantation. Patients with PH had a higher cumulative incidence of waitlist mortality and a lower cumulative incidence of retransplantation compared with patients without PH. Differences by PH status were significant for both waitlist mortality (Gray’s test p=0.037) and retransplantation (Gray’s test p=0.026).
